# Intestinal Microbiota Mediates High-Fructose and High-Fat Diets to Induce Chronic Intestinal Inflammation

**DOI:** 10.3389/fcimb.2021.654074

**Published:** 2021-06-16

**Authors:** Rong Tan, Huiwei Dong, Zhengshan Chen, Min Jin, Jing Yin, Haibei Li, Danyang Shi, Yifan Shao, Huaran Wang, Tianjiao Chen, Dong Yang, Junwen Li

**Affiliations:** Tianjin Institute of Environmental and Operational Medicine, Tianjin, China

**Keywords:** high-fructose diet, high-fat diet, intestinal inflammation, fecal output, metabolites, intestinal microbiota

## Abstract

An unhealthy diet has been linked to increased incidence of chronic diseases. To investigate the relationship between diet and intestinal inflammation, mice in two experimental groups were fed on a high-fat diet or high-fructose diet, respectively. The result showed that the defecation volume of the experimental groups was significantly reduced compared with that of the control group, and the levels of pro-inflammatory cytokines (interleukin (IL)-1β and IL-6) and IgG in serum were increased significantly. In addition, inflammatory cell infiltration was observed in intestinal tissue, indicating that a high-fructose or high-fat diet can lead to constipation and inflammation. Further analysis showed that the microbial composition of the experimental groups changed significantly, including a decrease of the *Bacteroidetes/Firmicutes* ratio and increased levels of *Bacteroides*, *Akkermansia*, *Lactobacillus*, and *Ruminococcus*, which might be associated with inflammation. The results of pro-inflammatory metabolites analysis showed that the levels of arachidonic acid, stearic acid, and indoxylsulfuric acid were significantly increased in the experimental groups, which were related significantly to *Bacteroides*, *Enterococcus*, and *Akkermansia*. Meanwhile, the content of 5-hydroxytryptamine (5-HT) was significantly decreased, which might cause constipation by reducing intestinal peristalsis. Moreover, transplantation of fecal bacteria from inflammatory mice caused constipation and inflammation in normal mice, which could be relieved by feeding a normal diet. The results of the present study indicated that changes in intestinal microbiota and microbial metabolites may underlie chronic intestinal inflammation and constipation caused by high-fructose and high-fat diets.

## Introduction

The intestinal microbiota is a complex and dynamic microbial ecosystem, whose composition is determined by genetic and environmental factors, among which diet is the most important factor that determines changes to the intestinal microbiota (Johnson et al., 2019). Zhang et al. reported that dietary changes could explain 57% of intestinal microbiota changes, while genetic mutations accounted for less than 12% (Zhang et al., 2010). In recent years, people have paid more attention to the influence of diet on intestinal microorganisms, and the mechanism might be that nutrients interact directly with the microbiota to promote or inhibit its growth (Bibbo et al., 2016). The ability to obtain energy from specific diets gives certain members of the gut microbial community a direct competitive advantage that increases their multiplication.

In humans, eating a diet consisting entirely of animal products results in the accumulation of bili-tolerant bacteria (*Alistipes*, *Bilophila*, and *Bacteroides*) and eating a diet consisting of plant products promotes the Firmicutes (*Roseburia*, *Eubacterium*, and *Ruminococcus*), which metabolize plant polysaccharides. In mice, regardless of the genotype studied, the intake of a high-fat diet (HFD) or high-fat high-sugar western diet (HFHSD) was associated with decreased Bacteroides levels and increased Firmicutes and Proteobacteria, in a dose-dependent manner (Hildebrandt et al., 2009). Once the intestinal microbiota balance is broken, an inappropriate inflammatory response might be triggered, resulting in host cell damage or autoimmune diseases. There is evidence that microbial malnutrition is associated with a variety of human diseases, including allergies, asthma, inflammatory bowel disease (IBD), irritable bowel syndrome (IBS), obesity, and cardiovascular disease (Wilson et al., 2019).

The intestinal microbiota plays an important role in the body’s metabolism, immunity, and nervous system development, and its abnormal structure or function might be an important cause of disease. Therefore, it has been called the “Second Genome”. In the context of complex environmental factors such as diet, the homeostasis between the intestinal microbiota and the mucosal immune system is easily disturbed (Kaplan, 2015). Intestinal metabolites mainly comprise the diet, modified metabolites, and microorganism-derived compounds. Metabolites of the intestinal microbiota, such as bile acid derivatives, short chain fatty acids (SCFAs), amino acid derivatives, and lipopolysaccharides, are important signals that can connect the intestinal microbiota with the host (Dorrestein et al., 2014). Therefore, the intestinal microbiota and its metabolites can interact with the host in different ways to affect homeostasis (Manichanh et al., 2012).

Some studies have reported that diet will change the genetic composition and metabolic activity of the microbes that live in our bodies. However, the relationship between the changes to the intestinal microbiota induced by high-fat and high-fructose diets and intestinal inflammation and constipation remain unclear. In the present study, to explore this relationship, Balb/c mice were used as model animals to observe the changes in proinflammatory cytokine expression and intestinal pathology under different dietary conditions, and to further analyze the changes to the intestinal microbiota and their metabolites. The results were further verified by the transplantation of fecal bacteria from inflamed mice into normal mice, and by feeding a normal diet to the inflamed mice. The results suggested that structural changes to the intestinal microbiota mediate the constipation and inflammation induced by high-fructose and high-fat diets, which provides a scientific basis to adjust dietary structures and promote health.

## Methods

### Animal Experiments

The experiment used 6–8 week old adult Balb/c male mice (Beijing Huafukang Biological Company, Beijing, China). The mice were fed freely and their weight was measured every week. The mice in the experimental groups were fed a high-fructose diet (60%) (Tain et al., 2018) or a high-fat diet (60 kcal%) (Tong et al., 2019), and the control group was fed a normal diet (Beijing Xiaoshuyoutai Biological Company, Beijing, China). There were 10 mice in each group of high-fructose diet experimental group, high-fat diet experimental group and normal diet control group. In the fecal bacteria transplantation (FBT) experiment, the high-fructose fecal bacteria transplantation group, the high-fat fecal bacteria transplantation group and the control group consisted of 10 mice in each group. In the normal diet recovery experiment, the changed high-fructose group, the changed high-fat group and the control group had 10 mice in each group. During the experiment, the fresh feces of the mice were collected within four hours in a sterile centrifuge tube, and immediately placed at −80°C and stored. Blood was collected through the tail tip of the mouse (Clemmensen et al., 2013), and about 100 μl of blood was collected in a 500 μl sterile centrifuge tube. After standing at room temperature for 4 h, the supernatant was collected after centrifugation at 1000 × *g* for 10 min and stored at −80°C. The mice were given 100 μl of the microbiota suspension four times a week for four weeks. To prepare the microbiota suspension, 2–5 fresh feces pellets (80–100 mg) were resuspended with vortexing in 600 μl of reduced phosphate buffered saline (PBS). After resuspension, the tubes containing the feces in reduced PBS were centrifuged at 500 × *g* for 1 min to remove insoluble material, and 100 μl of supernatant was administered to the mice by oral gavage (Barcena et al., 2019). At the end of the experiment, the animals were sacrificed by cervical dislocation, the abdominal cavity was opened, and the intestines were removed. After washing the intestinal contents with physiological saline, the intestinal tissue was placed in a centrifuge tube containing 4% paraformaldehyde for storage (Accogli et al., 2018). The Institutional Animal Care and Use Committees (IACUCs) of the Tianjin Institute of Environmental and Operational Medicine reviewed and approved all the experimental procedures, which were performed in accordance with the animal research guidelines of the Chinese Physiological Society. This study did not involve any endangered or protected animal species, nor did it cause unnecessary harm to laboratory animals.

### Inflammatory Cytokines and Intestinal Pathological Analysis

Serum inflammation-related cytokines, including interleukin 2 (IL-2), interleukin 6 (IL-6), immunoglobulin g (IgG) levels were determined using a microplate reader (Molecular Devices Corporation, San Jose, CA, USA.) and a cytokine detection kit (Jiangsu Enzyme Industry Co., Ltd., Jiangsu, China). These assays were based on the enzyme-linked immunosorbent assay (ELISA) technique (Pirim Gorgun et al., 2017). One cm of intestinal tissue was fixed using 4% paraformaldehyde, paraffin-embedded by dehydration, clearing, and wax-immersion according to standard procedures. The tissue was then cut into 5-μm-thick blocks and stained using H&E. The sections were viewed under a high power microscope (200×, 400×) (Zhu and Xie, 2018) to observe the pathological changes, such as epithelial edema, muscular layer injury, goblet cell number, and intestinal mucosa continuity (Teicher et al., 1963). Immunofluorescence staining was performed on 1 cm intestinal tissue fixed with 4% paraformaldehyde in accordance with standard procedures, such as paraffin sectioning, antigen repair, autofluorescence quenching, transparency, sealing, addition of primary and secondary antibodies, 4,6-diamidino-2-phenylindole (DAPI) re-staining and nuclear sealing. Finally, the immunofluorescence of paraffin-embedded sections was viewed under a high-power microscope (200×, 400×). The nuclei stained by DAPI were blue under ultraviolet excitation and cells positively stained for CD3 and CD4 appeared as red or green (Im et al., 2019).

### Fecal Bacterial Community Determination

The genomic DNA of fecal samples was extracted using the cetyltrimethylammonium bromide (CATB) method (Gomez-Acata et al., 2019), and then the purity and concentration of the DNA were detected using agarose gel electrophoresis. The diluted genomic DNA was used as a template for PCR amplification. The obtained PCR products were detected by electrophoresis through 2% agarose gels, and the target bands were recovered using a gel recovery kit from Qiagen (QIAGEN, Hilden, Germany). A DNA PCR-free Sample Preparation Kit (TruSeq at Illumina, San Diego, CA, USA) was used for library construction. After Qubit and quantitative real-time PCR (qPCR) quantification, the quality of the constructed library was determined, and then the library was sequenced on a NovaSeq6000 Illumina system. The original data was spliced using FLASH (v1.2.11, http://ccb.jhu.edu/software/FLASH/) (Magoc and Salzberg, 2011) to get Raw Tags. Then, clean tags were obtained by strict filtering of the original data (Bokulich et al., 2013). The quality control process of Qiime (v2.0, http://qiime2.org/) (Bokulich et al., 2018; Bolyen et al., 2019) was used. The tag sequences were compared with the species annotation database and the chimera sequences were removed to obtain the final effective tags (https://github.com/torognes/vsearch/) (Rognes et al., 2016). Uparse software (v7.0.100, http://www.drive5.com/uparse/) (Haas et al., 2011) was used to cluster all the effective tags of all samples. By default, the sequences are clustered into operational taxonomic units (OTUs) with 97% identity. Species annotation of OTU sequences was performed using the Mothur method and the SILVA138 (http://www.arb-silva.de/) (Edgar, 2013) SSUrRNA database (Wang et al., 2007) (with a threshold of 0.8 ~ 1), to obtain the taxonomic information at each classification level: Kingdom, phylum, class, order, family, genus, and the species count for the community composition of each sample was determined. Finally, the data of each sample was normalized, and the data with the smallest amount of data in the sample was used as the standard. Alpha diversity analysis and Beta diversity analysis were based on the normalized data.

### Determination of Fecal Metabolites

Metabolite extraction used 100 mg of fecal sample, which was placed in a centrifuge tube, and 500 μl of 80% methanol aqueous solution containing 0.1% formic acid. The sample was vortexed and left to stand in an ice bath for 5 min, before being centrifuged at 15000 × *g*, at 4°C for 10 min. The supernatant was diluted with mass spectrometry solution to a 53% methanol content, and centrifuged at 15000 × *g* at 4°C for 10 min. The supernatant was collected and injected into an LC-MS instrument (Mahmud et al., 2017) for analysis. Equal volumes of samples from each experimental sample were taken and mixed as QC samples. The blank sample was a 53% methanol aqueous solution containing 0.1% formic acid instead of the experimental sample. The off-machine data was imported into the CD search software 1 (CD3.1, Thermo Fisher Scientific, Waltham, MA, USA), and simple screening of the retention time, mass-to-charge ratio, and other parameters was performed. Then, different samples were peak aligned according to a retention time deviation of 0.2 min and a mass deviation of 5 ppm to make the identification more accurate. The data were then processed according to a set mass deviation of 5 ppm, a signal strength deviation of 30%, a signal-to-noise ratio 3, a minimum signal strength of 100000, plus Peak extraction with ion and other information. At the same time, the peak area was quantified, the target ion was integrated, and then the molecular formula was predicted using the molecular ion peak and fragment ion. The metabolite date were annotated using the KEGG database (http://www.genome.jp/kegg/), HMDB database (http://www.Hmdb.ca/), and Lipidmaps database (http://www.lipidmaps.org/). The blank sample was used to remove background ions, and the quantitative results were normalized. Finally, the data identification and quantitative results were obtained. Metabolic pathways were analyzed using KGML (https://www.kegg.jp/kegg/xml/), version v0.7.2 DTD (Kanehisa and Goto, 2000; Kanehisa, 2019; Kanehisa et al., 2020).

### Statistical Analysis

Except for Mann-Whitney u-test calculation using SPSS 16.0 (IBM Corp. Armonk, NY, USA), the rest were analyzed using Prism v3.02 and v5.01 (GraphPad Inc., La Jolla, CA, USA). For each group of mice, body weight, fecal output, and immune factors were compared using unpaired t tests. Dunnett-t test was used for multiple comparisons between the experimental groups and the control group. The statistical significance was set at p < 0.05, with p > 0.05 being not statistically significant. The significance of the pairwise comparison was expressed as: * p < 0.05; ** p < 0.01; *** p < 0.001. Sample complexity analysis (Alpha Diversity) use the Qiime software to calculate Chao1 and Shannon index. The difference analysis between Alpha diversity index groups was conducted with parametric and non-parametric tests, respectively. The experiment was larger than two groups; therefore, Tukey’s test and Wilcox’s test were used. Multi-sample comparison analysis (Beta Diversity) used the Qiime software to calculate Unifrac distance and to build the unweighted pair group method with arithmetic mean (UPGMA) sample clustering tree. PCoA analysis used the R software’s WGCNA, stats, and ggplot2 software package; and NMDS analysis uses the R software’s vegan software package. The R software was used to analyze the differences among the Beta diversity index groups, and the parametric test and non-parametric test were conducted, respectively. LAnosim, MRPP, and Adonis analysis used the anosim function, mrpp function, and adonis function of the R vegan package respectively. AMOVA analysis used the Mothur software’s amova function (Lai et al., 2020).

## Results

### High-Fructose and High-Fat Diets Change the Body Weight and Fecal Volume of Mice

**Figure 1:**
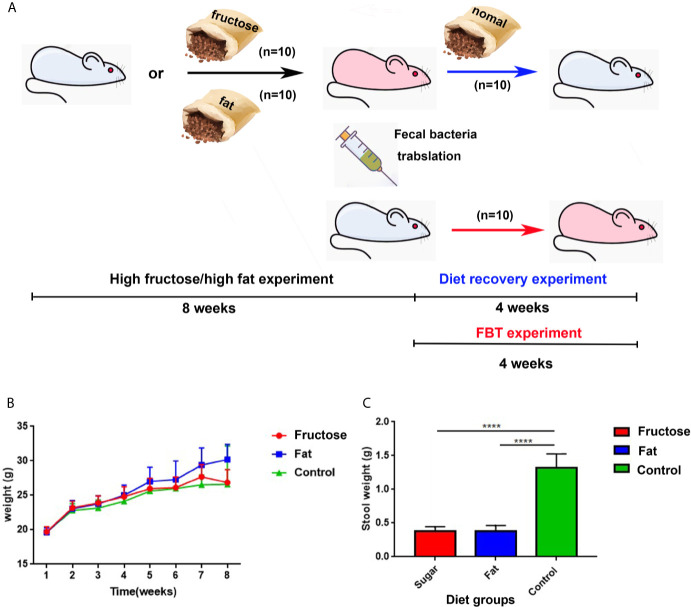
The experimental design and animal experiment flow chart and changes in body weight and fecal volume of mice. Blue mice represent healthy mice, and pink mice represent inflamed mice. The black arrow indicates the diet-induced inflammation experiment, the red arrow indicates the fecal bacteria transplantation experiment, and the blue arrow indicates the diet recovery experiment **(A)**. Changes in body weight of mice at 8 weeks **(B)**. 24 h fecal volume of mice in each group **(C)**. (****p < 0.0001).

The flow chart of the experimental design and treatment to animals is shown in **Figure 1A**. Balb/c mice were fed freely with three diets. The results showed that compared with the control group fed with a normal diet, the high-fructose group showed a significant difference in body weight only at the third week (p = 0.0364) and the seventh week (p = 0.0151). There was significant difference in body weight between the high-fat group and the control group from the fourth week to the eighth week (p < 0.05, **Figure 1B**). The excretion of fecal material by the mice in the experimental groups was significantly lower than that of the control group. Generally, a normal adult mouse excretes about 1.2–2.4 g of feces per day (the amount of water that evaporates from the feces during collection is negligible). The control group in this experiment excreted about 1.25 g, which was within the normal range. The high-fructose group and high-fat group excreted about 0.4 g, only one third of that of the control group, and the difference was significant (p < 0.0001, **Figure 1C**).

### High-Fructose and High-Fat Diets Induces Intestinal and Systemic Inflammation

**Figure 2:**
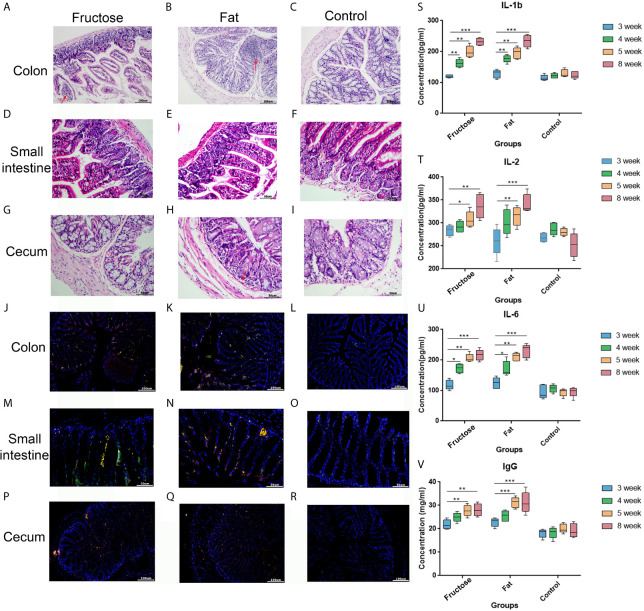
Effects of high-fructose and high-fat diets on intestinal inflammation. The colon pathological sections were from the high-fructose group **(A, J)**, the high-fat group **(B, K)** and the control group **(C, L)**. The small intestine pathological sections were from the high-fructose group **(D, M)**, the high-fat group **(E, N)** and the control group **(F, O)**. The cecum pathological sections were from the high-fructose group **(G, P)**, the high-fat group **(H, Q)**, and the control group **(I, R)**. In H&E staining images, the nucleus is blue and the cytoplasm is red; the red arrow indicates inflammatory cell infiltration. In the immunofluorescence staining images, the nuclei stained by DAPI are blue under UV excitation, the cells labeled for CD3 are red, and those labeled for CD4 are green. Cells expressing both CD3 and CD4 appear yellow after superimposition. The levels of IL-1 β **(S)**, IL-2 **(T)**, IL-6 **(U)**, and IgG **(V)** were measured from the 3rd week to the 8th week. (*p < 0.05, **p < 0.01, ***p < 0.001).

In the high-fructose group, the overall structure of the colon and cecum was abnormal. The intestinal mucosal layer was necrotic and degenerated, and some epithelial cells had been shed. The tissues showed obvious infiltration of inflammatory cells (**Figures 2A, G**). The overall structure of the small intestine was abnormal, but there was no obvious inflammatory cell infiltration in the tissues (**Figure 2D**). In the high-fat group, the structure of the colon and small intestine and cecum was abnormal. The intestinal mucosal epithelial cells degenerated and died, the cells were loosely arranged, and there was a protein exudate. Inflammatory cell infiltration was observed in these tissues (**Figures 2B, E, H**). In the normal diet control group, the overall structure of the intestinal tissue was normal, and the tissue showed no obvious inflammatory cell infiltration (**Figures 2C, F, I**). This suggested that high-fructose and high-fat diets can induce inflammation in different intestinal segments of mice.

In the immunofluorescence staining analyses, the nuclei stained with DAPI were blue under ultraviolet excitation, and the cells labeled for CD3 and CD4 were red and green, respectively. When these three images were superimposed, the inflammatory cells showed yellow dots. The high-fructose group (**Figures 2J, M, P**) and high-fat group (**Figures 2K, N, Q**) had more yellow spots, while the control group had almost none (**Figures 2L, O, R**). The results of immunofluorescence were consistent with those of the hematoxylin and eosin (H&E) staining.

In addition, each intestinal segment was scored to evaluate the inflammatory intestinal morphology in mice quantitatively. Inflammatory cell infiltration, epithelial changes, and mucosal structure of the intestinal sections were scored, respectively (Erben et al., 2014). The results showed that inflammatory cell infiltration was found in the intestines of the high-fructose group and high-fat group (score ≥ 1), and the mucosal and epithelial structures were changed (score ≥ 2). In the control group, there was no obvious inflammatory cell infiltration (score = 0) and the mucosa and epithelium were relatively intact and normal (score ≤ 1). Combined with the scores of the three parts, the total score of the experimental groups was greater than or equal to 4 points, while that of the control group was less than or equal to 2 points (**Table 1**).

**Table 1:** Histomorphological scores of intestinal inflammation in mice.

Tissue	Sample number	Inflammatory cell infiltration score	Epithelial change score	Mucosal structure score	Total score
Colon	A	2	3	2	7
	B	2	3	3	8
	C	0	1	1	2
Small intestine	D	0	2	2	4
	E	1	2	2	5
	F	0	0	0	0
Cecum	G	0	2	2	4
	H	2	2	2	6
	I	0	1	0	1

(1) Inflammatory cell (infiltrate severity): Minimal: 1, Mild: 2, Moderate: 3.

(2) Epithelial changes (Goblet cell loss): Minimal: 1–2, Mild: 2–3, Moderate: 3–4.

(3) Mucosal architecture (Villous blunting): Mild: 1–3, Moderate: 2–4, Villous atrophy: 3–5.

Enzyme-linked immunosorbent assays (ELISAs) showed that interleukin (IL)-1β, IL-2, and IL-6 levels in the two experimental groups were significantly higher than those in the control group after 8 weeks of high-fructose and high-fat feeding (p < 0.01, **Figures 2S–U**). In addition, serum immune factors, e.g., immunoglobulin G (IgG), levels in the two experimental groups were also significantly higher than those in the control group (p < 0.01, **Figure 2V**). Serum immune factors were identified at 3, 4, and 5 weeks before the stable formation of inflammation, and the levels of these pro-inflammatory factors gradually increased from the fourth week, becoming significantly higher than those in the control group. There was no statistical difference between the results in week 5 and week 8, indicating that inflammation had formed by week 5. These results suggested that a high-fructose or high-fat diet might induce systemic inflammation in mice.

### High-Fructose and High-Fat Diets Change the Intestinal Microbiota in Mice

**Figure 3:**
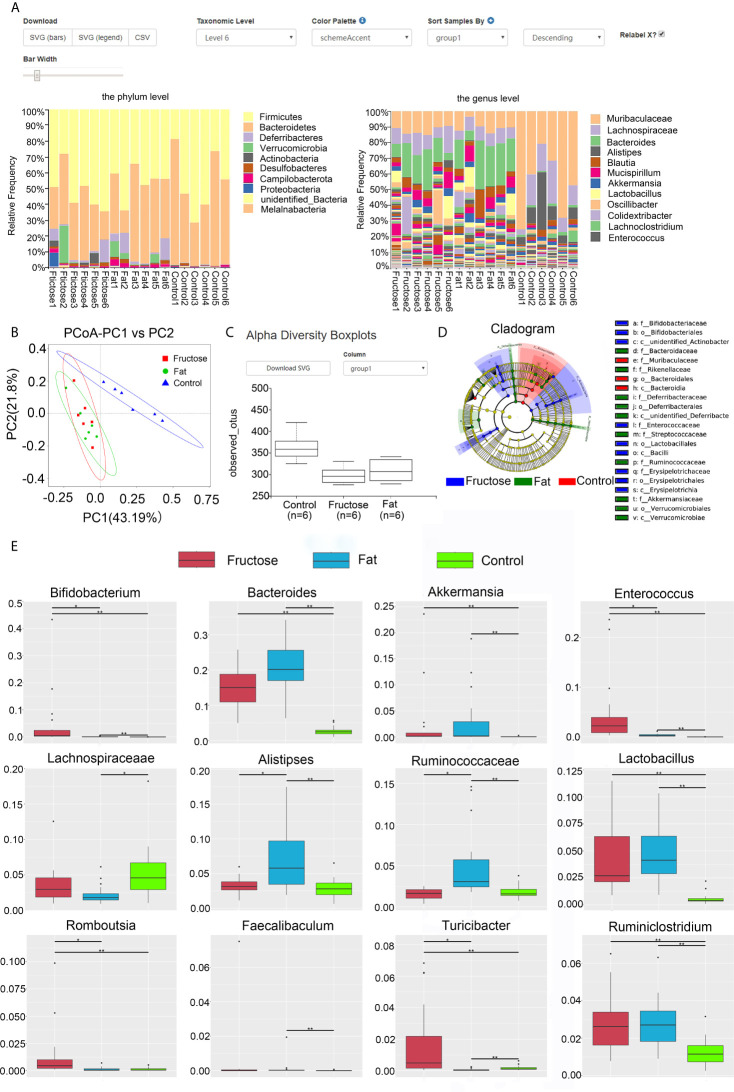
High-fructose and high-fat diets changes the intestinal microbiota in mice. The relative abundance of the top 10 microbes at the phyla level and genus level in each group is shown in the histogram, with the horizontal axis being the sample name and the vertical axis being the relative abundance **(A)**. In the PCoA analysis, the abscissa represents one principal component, the ordinate represents another principal component, and the percentage represents the contribution value of principal component to the sample difference **(B)**. Alpha diversity box chart: Observed species is the observed number of species (also known as the OTU number) **(C)**. An evolutionary branching diagram in which the circles radiating from the inside to the outside represent the taxonomic level from phylum to genus (or species) **(D)**. The abundance distribution box diagram of different species between groups, in which the horizontal axis is the sample grouping and the vertical axis is the relative abundance of the corresponding species **(E)**. (* p < 0.05, **p < 0.01).

At the phylum level, comparing the relative abundance in each diet group (**Figure 3A**) we found that the ratio of Bacteroidetes/Firmicutes in the control group was the highest, followed by the high-fat group, and was lowest in the high-fructose group. Both the high-fat group and the high-fructose group had higher abundances of *Deferribacteres* (high-fat, 3.76%; high-fructose, 3.1%) and *Verrucomicrobia* (high-fructose, 2.42%; high-fat, 2.91%) compared with those in the control group. *Actinobacteria* (5.27%) in the high-fructose group had a higher abundance, while *Proteobacteria* (1.43%) in the high-fat group had a higher abundance compared with those in the control group. At the genus level, the two experimental groups had higher abundances of *Bacteroides*, *Akkermansia*, *Alitipes*, and *Mucispirillum* than the control group. In addition, the high-fructose group had higher abundances of *Bifidobacterium* and *Enterococcus* than the control group. The high-fat group had a higher abundance of *Ruminococcaceae* than the control group.

Through principal coordinates analysis (PCoA), we found that the samples from high-fructose group and high-fat group had certain similarity, with some samples being located in the same area of the plot; however, there were significant differences compared with the samples from the control group (**Figure 3B**). By analyzing the alpha diversity of a single sample, we found that the total number of species from high to low was, in order: The control group, the high-fat group, and the high-fructose group (**Figure 3C**). According to the Shannon-Wiener diversity index, the community diversity, from high to low, was as follows: The control group, the high-fructose group, and the high-fat group. The Wilcox rank sum test analysis of these data showed that the difference between the high-fructose group and the control group was significant (p < 0.01), as was the difference between the high-fat group and the control group (p < 0.01). LEfSe (Linear discriminant analysis effect size) analysis showed that the biomarkers of the high-fructose group were *Bifidobacterceae*, *Enterococcaceae*, and *Erysipelotrichaceae*. The biomarkers of the high-fat group were *Bacteroidaceae*, *Rikenellaceae*, *Deferribacteraceae*, *Streptococcaceae*, *Ruminococcaceae*, and *Akkermansiaceae* (**Figure 3D**). Metastat analysis showed that the levels of *Bifidobacterium*, *Bacteroides*, *Akkermansia*, *Enterococcus*, *Lactobacillus*, and *Ruminiclostridium* in the two experimental groups were significantly higher than those in the control group (p < 0.05). In addition, levels of *Rombousia* and *Turicibacter* in the high-fructose group were significantly higher than those in the control group (p < 0.05). The level of *Ruminococcaceae* in the high-fat group was significantly higher than that in the control group (p < 0.05). The above results indicated that the high-fructose diet and high-fat diet could change the structure and abundance of the intestinal microbiota of mice significantly (**Figure 3E**).

### High-Fructose and High-Fat Diets Lead to Changes to Metabolites in Mice

**Figure 4:**
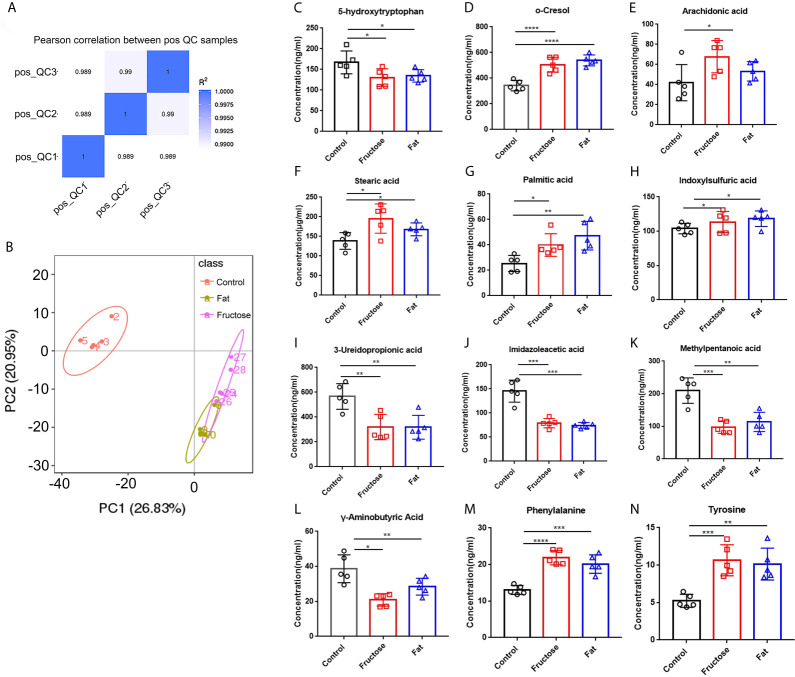
The effect of high-fructose and high-fat diets on metabolites. The Pearson correlation coefficient between the quality control (QC) samples was calculated based on the peak area value **(A)**. In the PCoA analysis, the abscissa PC1 represents the first principal component score, and the scattering of different colors represent the samples of the different experimental groups. The closer the distance is, the smaller the difference between the samples is, and the ellipse is the 95% confidence interval **(B)**. The contents of o-cresol **(C)**, arachidonic acid **(D)**, stearic acid **(E)**, palmitic acid **(F)** and indole sulfuric acid **(G)** in fecal metabolites were determined. The contents of SCFAs in fecal metabolites included 3-ureopropanoic acid **(H)**, imidazole acetic acid **(I)**, methylvaleric acid **(J)**, and γ-aminobutyric acid **(K)**. The content of amino acids in fecal metabolites, including phenylalanine **(L)**, tyrosine **(M)**, and 5-hydroxytryptophan **(N)**. (*p < 0.05, **p < 0.01, ***p < 0.001, ****p < 0.0001).

In the correlation analysis of the quality control (QC) samples, a higher correlation (the closer R^2^ is to 1) indicates better stability of the entire detection process and higher data quality. In this measurement, R^2^ was close to 1, indicating high data quality (**Figure 4A**). In the principal component analysis (PCA) (Wen et al., 2017), the smaller the difference in samples between each group, the more samples will aggregate in a certain range. Parts of the samples from the high-fructose group and overlapped with those from the high-fat group (**Figure 4B**). PERMANOVA test results showed that there were significant differences in clustering between the groups (p < 0.05, **Table 2**).

**Table 2:** The result of the PERMANOVA test.

Group	Df	Sums of squares	Mean squares	F. Model	Variation (R^2^)	Pr
Control/Fat	1	0.237587	0.237587	8.297696	0.509133	0.01
Control/Fructose	1	0.177126	0.177126	7.343092	0.478593	0.01
Fat/Fructose	1	0.114027	0.114027	6.691307	0.45546	0.011

Df: degree of freedom.

R^2^: The explanation degree of different groups to the sample difference. The larger the R^2^, the higher the group’s explanation of the difference.

Pr: P value, less than 0.05 shows that the reliability of this test is high.

The analysis of different metabolites showed that the levels of some pro-inflammatory metabolites, such as cresol, arachidonic acid, stearic acid, palmitic acid, and indole sulfuric acid in the high-fructose and high-fat groups were significantly higher than those in the control group (p < 0.05) (**Figures 4C–G**). In addition, the levels of 25 short chain fatty acids (SCFAs) in the high-fructose group and the 23 SCFAs in the high-fat group were significantly different from those in the control group. Among them, the concentrations of SCFAs in the high-fructose and high-fat groups, such as acetic acid, propionic acid, butyric acid, and pentanoic acid, were significantly lower than those in the control group (p < 0.05), which would cause intestinal cells to lose protection, resulting in intestinal inflammation (**Figures 4H–K**). The amino acid levels of the two experimental groups were also significantly different from those of the control group, such as the levels of phenylalanine, tyrosine, leucine, isoleucine, and valine (p < 0.05). In the high-fructose group, the levels of 20 amino acids were significantly different from those of the control group. In the high-fat group, the levels of 16 amino acids were significantly different compared with those from the control group. Among them, phenylalanine and tyrosine levels were significantly lower than those in the control group (**Figures 4L, M**). In addition, the 5-hydroxytryptophan content of the two experimental groups was significantly lower than that of the control group, which would reduce bowel movements and cause constipation (**Figure 4N**). The above results indicated that high-fructose and high-fat diets can cause changes in a variety of metabolites, ultimately leading to inflammation and constipation.

Correlation analysis was performed between the significantly different genera identified through 16S sequencing and the significantly different metabolites. Indoxyl sulphate and palmitic acid, which were associated with inflammation, were significantly related to *Lactobacillus*, *Bacteroides* and *Peptococcus*. There was a significant correlation between the high-abundance bacteria and pro-inflammatory metabolites. For example, arachidonic acid, stearic acid, and indoxylsulfuric acid correlated positively with *Bacteroides*, *Enterococcus*, and *Akkermansia*. This indicated that the intestinal microbiota and their metabolites formed by a high-fructose and high-fat diet are the key factors leading to intestinal inflammation and constipation.

### Fecal Bacteria Transplantation Induced Constipation and Inflammationin Normal Mice

**Figure 5:**
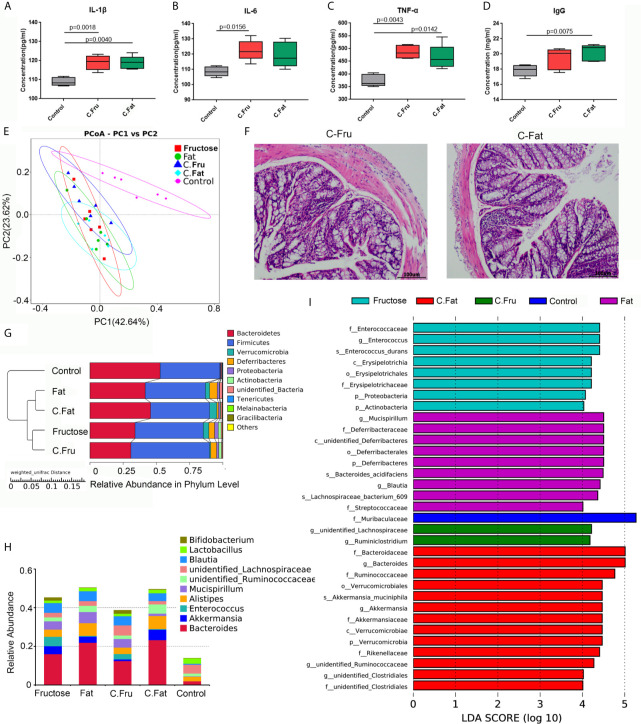
Fecal bacteria transplantation changes the intestinal microbiota and induces inflammation. Serum immune factors, including IL-1 β **(A)**, IL-6 **(B)**, TNF–α **(C)**, and immunoglobulin IgG **(D)** associated with inflammation. In the PCoA analysis, the abscissa and ordinate, respectively, represent a principal component, and percentage represents the contribution value of the principal component to the sample difference **(E)**. H&E staining showing the intestinal state of the experimental group after transplantation of fecal bacteria **(F)**. The UPGMA cluster tree, on the left is the UPGMA cluster tree structure, and on the right is the relative abundance distribution map of species at the phylum level **(G)**. Histogram of species distribution at the genus level **(H)**. The histogram of LDA value distribution shows the species with significant differences in abundance among the different groups **(I)**. (C.Fru: mice transplanted with fecal bacteria from the high-fructose group, C.Fat: mice transplanted with fecal bacteria from the high-fat group).

The feces of mice with inflammation caused by the high-fructose and high-fat diet were treated by centrifugation and transplanted into normal mice *via* oral gavage. After 4 weeks, the serum inflammatory factor results showed that the levels of IL-1β (p = 0.0018), IL-6 (p=0.0156) and TNF-α (p=0.0043) in mice transplanted with fecal bacteria of high-fructose group (C.Fru) were significantly higher than those in the control group, and IgG difference was not significant. Moreover, the levels of IL-1β (p=0.0040), TNF-α (p=0.0142) and IgG (p=0.0075) in mice transplanted with fecal bacteria of the high-fat group (C.Fat) were significantly higher than those in the control group, while IL-6 was not significantly different (**Figures 5A–D**). Pathological analysis showed that the mice transplanted with fecal bacteria had abnormal tissue structure, obvious epithelial cell death, and inflammatory cell infiltration (**Figure 5F**). The amount of feces in the mice transplanted with fecal bacteria was significantly lower than that in the control group, which indicated that the bacteria and their contents in the feces could lead to intestinal inflammation and constipation.

The results of the mouse gut microbiota sequencing in the fecal bacteria transplantation experiment showed that four weeks after fecal bacteria transplantation, the intestinal microbiota of the two groups changed significantly. The PCoA results showed that the intestinal microbial community structure of the mice transplanted with fecal bacteria from the high-fructose group was similar to that of the high-fructose group. The intestinal microbial community structure of mice transplanted with fecal bacteria from the high-fat group was similar to that of the high-fat group (**Figure 5E**). An unweighted pair group method with arithmetic mean (UPGMA) clustering tree was constructed by clustering the samples of all groups. The result showed that the fructose fecal bacteria transplantation group and the high-fructose group were closest and could be grouped together. The fat fecal bacteria transplantation group was closest to the high-fat group, and could be grouped into one category. However, these four groups are far away from the control group and could not be clustered directly (**Figure 5G**).

Compared with the control group, *Bacteroides*, *Alistipes*, *Enterococcus*, *Bifidobacterium*, *Akkermansia* and *Blautia* levels were increased significantly in the fructose fecal bacteria transplantation group, which was consistent with the difference in the high-fructose diet group. Compared with the control group, there were significant differences in the levels of *Bacteroides*, *Alistipes*, *Lactococcus*, *Akkermansia*, *Blautia*, and *Ruminococcaceae* in the fat fecal bacteria transplantation group, which was consistent with the difference in the microbiota in the high-fat diet group (**Figure 5H**). LEfSe analysis showed that there were significant differences in biomarkers between the groups. The biomarkers of the high-fructose fecal bacteria transplantation group were *unidentified_Lachnospiraceae* and *Ruminiclostridium*, and the biomarkers of the high-fat fecal bacteria transplantation group were *Bacteroides*, *Akkermansia*, and *unidentified_Clostridiales* (**Figure 5I**). The above results showed that although the microbiota of the fecal bacteria after transplantation tended to be consistent with the original microbiota, there were still some differences. However, most of the altered microbiota could play a major role in promoting inflammation and constipation. It can be inferred that the gut microbiota induced by high-fructose and high-fat diet can change the gut microbiota and its functions in normal mice.

### Normal Diet Can Relieve Constipation and Inflammation in Mice

**Figure 6:**
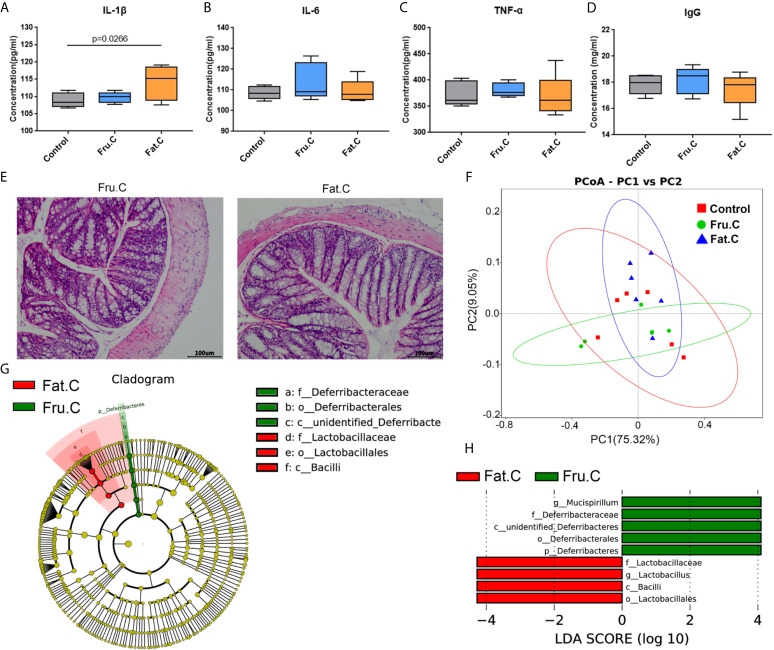
Diet changes the intestinal microbiota and then affects intestinal inflammation. Levels of proinflammatory serum immune factors, including IL-1β **(A)**, IL-6 **(B)**, TNF–α **(C)**, and immunoglobulin IgG **(D)** associated with inflammation. H&E staining showed the intestinal state of the experimental group after transplantation of fecal bacteria **(E)**. Based on the unweighted UniFrac distance PCoA analysis **(F)**. In the cladogram, the circles radiating from inside to outside represent the taxonomic level from phylum to genus (or species). Each small circle at different taxonomic levels represents a taxonomic level, and the diameter of the small circle is proportional to the relative abundance **(G)**. The histogram of LDA value distribution shows the species whose LDA score is greater than the set value (the default setting is 4); biomarkers with statistical differences between groups **(H)**. (Fru.C, high-fructose diet changed to normal diet group; Fat.C, high-fat diet changed to normal diet group).

The mice with inflammation caused by the high-fructose and high-fat diets were fed a normal diet for four weeks. The results of intestinal pathology and serum inflammatory markers showed that the intestinal structure of the experimental mice had returned to normal, and there was no obvious inflammatory cell infiltration and cell necrosis (**Figure 6E**). In the diet change groups, the inflammatory factors had no significant difference compared with the normal diet group and were significantly lower than those before diet change (**Figures 6A–D**), which suggested that the inflammation of mice had been relieved. After 1–2 days of diet change, the amount of feces produced by the mice increased rapidly. There was no significant difference between the changed high-fructose group (Fru.C) and the changed high-fat group (Fat.C) and the control group. This showed that diet plays a major role in this process, and a continuous normal diet can alleviate the symptoms of constipation and inflammation in the body.

After 4 weeks of the normal diet, the results of the intestinal microbiota sequencing of mice showed that there was no significant difference between the changed high-fructose group, changed high-fat group, and the control group. PCoA analysis showed that the sample aggregation of the changed high-fructose group and the changed high-fat group was close to that of the control group, indicating that there was a high similarity in species composition among the three groups (**Figure 6F**). LEfSe analysis showed that although the community structure of the changed high-fructose group and the changed high-fat group were similar, there were still some different species in the intestinal microbiota under different diets. *Mucispirillum* in the changed high-fructose group and *Lactobacillus* in the changed high-fat group are the dominant members of the microbiota respectively, which indicated that because of the previous long-term diet habits, even in the case of diet changes accompanied by dramatic changes in intestinal microbiota, some of the dominant bacteria formed by previous dietary effects will be retained (**Figures 6G, H**). The above results showed that when the diet returns to normal, the intestinal microbiota will respond quickly to adapt to the new diet mode and coexist with it, which can also maximize the survival rate of the bacterial community. Recovery of the microbiota will be followed by recovery of digestive and metabolic function, ultimately relieving inflammation and constipation symptoms.

## Discussion

Diet is an essential factor for the growth and development of the body, providing energy support for the normal operation of various bodily functions. However, diet components might also affect the host-microbial community interaction, resulting in changes in the intestinal microbiota structure, thus adversely affecting the host. High-fructose and high-fat diets can provide sufficient nutrients to increase the number of Bacteroides and Lactobacilli, which can synthesize complex digestive enzymes to degrade the carbohydrates that are not completely digested by upstream processes (Jost et al., 2015; Rakoff-Nahoum et al., 2016). The carbohydrates in the diet are completely digested, producing a large number of metabolites, which include indoxylsulfuric acid, arachidonic acid, and stearic acid. These pro-inflammatory metabolites can signal to the gut to cause changes in tight junction proteins, thus increasing intestinal permeability and constantly stimulate the intestinal mucosal immune system to produce inappropriate immune response eventually leading to the occurrence of intestinal inflammation. In the inflammatory state, intestinal absorption function will be weakened, so that the metabolites cannot be absorbed effectively and utilized, resulting in constipation. Constipation can make metabolites accumulate further, eventually aggravating inflammation. Transplantation of fecal bacteria from inflamed mice to normal mice can also lead to changes to the intestinal microbiota, and then induce constipation and inflammation. This indicates that diet-induced gut microbiota can also change the structure and function of normal mice’s gut microbiota. In addition, after high-fructose and high-fat diets induce constipation and inflammation in mice, feeding a normal diet can quickly change the microbiota and further alleviate these symptoms. This indicates that after the normal diet restores the microbiota, its digestive and metabolic functions will also be restored, ultimately alleviating inflammation and constipation symptoms.

The pro-inflammatory cytokines IL-2 and IL-6, and IgG were significantly increased in the experimental groups. IL-2 is a cell growth factor in the immune system. It can regulate the activity of white blood cells in the immune system, promote the proliferation of T-helper cell 0 (Th0) and cytotoxic T lymphocytes (CTLs), and also participates in antibody responses. IL-6 can induce B cell differentiation and antibody production, and induce T cell activation, proliferation, and differentiation (Rose et al., 2001). IL-6 participates in the body’s immune response (Abbas et al., 2018) and is an initiator of inflammation. IgG is the main component of immunoglobulin and makes up approximately 70–80% of total antibody count in serum (Hamano et al., 2001). Increased IgG in serum is usually accompanied by various inflammations in the body. The normal reference value of serum IgG content is 7.0–17.0 mg/mL, and it could be seen from the test results that the control group maintained the normal level, while the IgG levels in the experimental groups were much higher than the normal value. A high-fat diet induces colonic inflammation, including increased expression of pro-inflammatory cytokines, induction of toll-like receptor 4 (TLR4), iNOS, COX-2 and the activation of NF-kB in the colon. In addition, intestinal tissues express higher levels of pro-inflammatory cytokines, including TNF-a, IL-1 and IL-6 (Weisberg et al., 2003). A high-fat diet will also reduce the expression of claudin-1 and occludin, which are related to the tight junctions of the colon, resulting in increased intestinal permeability, suggesting that a high-fat diet will cause changes in the integrity of the intestinal barrier (de La Serre et al., 2010). Therefore, lipopolysaccharide in the lumen may enter the intestinal lamina propria, where macrophages produce pro-inflammatory cytokines that enhance local inflammation (Neuman, 2007).

The analysis of the intestinal microbiota structure showed that the experimental groups were significantly different from the control group, mostly related to *Bacteroides*. In the process of intestinal metabolism, *Bacteroides* usually secrete surface glycoside hydrolase to digest polysaccharides extracellularly (Cartmell et al., 2018). The polysaccharide utilization of *Bacteroidetes* could be induced by the monomers or oligomers of the polysaccharides used. Large expansion of certain *Bacteroidetes* in the intestinal tract caused by the diet will lead to large proliferation of intraepithelial lymphocytes (IELs) and eventually, an increase in the IL-6 level (Kuhn et al., 2018), which is also consistent with the results of serum inflammatory factors. Mucus is synthesized and secreted by host goblet cells and forms an integral structural component of the mammal intestine. Its major function is to protect the intestinal epithelium from damage caused by food and digestive secretions. However, various anaerobic bacteria species of the gut microbiota, such as *Akkermansia*, *Bacteroides*, *Bifidobacterium*, and *Ruminococcus*, can use their specific enzyme activities to degrade mucin (Sicard et al., 2017). In addition, the high-fructose diet increased the intestinal permeability because of changes to tight junction proteins caused by signals in the intestines (Do et al., 2018). Destruction of the integrity of the intestinal barrier increases the chances of various metabolites contacting the immune system, which will ultimately increase the risk of inflammation. A diet rich in fat also induces intestinal tissue macrophage infiltration and an increase in pro-inflammatory cytokines, leading to an inflammatory response (Kim et al., 2012).

High-fructose and high-fat diets are usually associated with obesity. In the microbiome of obese individuals, carbohydrate metabolism-related pathways, such as fructose and mannose metabolism, galactose metabolism, starch and sucrose metabolism are highly enriched (Liu et al., 2017). Analysis of the metabolic pathways in the experimental groups showed that the pathways related to carbohydrate metabolism were highly enriched, indicating that the microbiota in the experimental groups had a stronger ability to utilize carbohydrates. Wan et al. (2019) showed that under the effect of a high-fat diet, the serum concentrations of phenylalanine, tyrosine, leucine, and valine in obese individuals were significantly higher than those in the control group. These amino acids correlated positively with homeostatic model assessment of insulin resistance (HOMA-IR), hyperglycemia, hyperlipidemia, and circulating inflammatory factors. In the present study, the concentration of most SCFAs were significantly lower in high-fructose and the high-fat diet group than in the control group. In addition, the levels of cresol, indole sulfuric acid, stearic acid, phenylalanine, tyrosine, leucine, and other inflammation-related metabolites in the feces of the high-fructose and high-fat groups were significantly higher than those of the control group. The 5-hydroxytryptophan level in the experimental group was significantly lower than that in the control group. It was reported that 5-hydroxytryptophan, as a marker of constipation, correlates negatively with constipation (Cao et al., 2017).

Most of the digestive and absorption functions of the intestines occur in the duodenum and small intestine, and are promoted by the long villi and microvilli, which contain enzymes that mediate the digestion and transport of nutrients (Wlodarska et al., 2015). The remaining indigestible nutrients will enter the colon for further digestion and absorption, and the unused remaining residues will be removed from the body. Inflammatory mucosal tissue in the intestines of patients presenting with Crohn’s disease (CD) and ulcerative colitis (UC) showed reduces inhibitor of apoptosis protein (IAP) production, which may occur through enhanced TLR4 signaling and increased bacterial translocation into the mucosa (Goldberg et al., 2008). Inflammatory diseases that affect the small intestine often result in reduced villi function, leading to malabsorption and malnutrition (Dewar and Ciclitira, 2005). The colon is also the place where microbial communities are degraded by enzymes and SCFAs are produced. SCFAs include acetate, propionate, and butyrate, which have a protective effect on epithelial cells and stimulate fluid absorption. UC can cause changes in the microbial composition and reduce the production of SCFAs, such that treatment with SCFAs can be clinically beneficial (Parada Venegas et al., 2019). In UC, butyric acid is beneficial, induces the regulation of T cell differentiation, and is critical to the resolution of inflammation through G-protein-coupled receptor signaling (Furusawa et al., 2013). Overgrowth of microorganisms might lead to changes in intestinal pathology, especially weakened villi, increased intestinal permeability, and chronic inflammation, which damages the proximal small intestine, and subsequently reduces nutrient absorption (Ngure et al., 2014). This may be an important cause of constipation.

Through the above analysis, we concluded that the intestinal microbiota disorder and metabolic characteristic changes caused by a high-nutrient diet will affect the integrity of the intestinal barrier and cause intestinal inflammation. However, as a result of intestinal inflammation, the body will increase intestinal permeability and cannot effectively absorb nutrients, such that nutrients are further accumulated, leading to constipation. These metabolites will continue to stimulate the intestinal mucosal immune system and eventually increase intestinal inflammation. Transplanting fecal bacteria from mice with inflammation and constipation into normal mice can lead to the recurrence of inflammation and constipation, and restoring normal diet can reduce the symptoms of inflammation and constipation. Therefore, long-term consumption of high-fat or high-fructose diet might lead to constipation and then cause intestinal inflammation; however, the recovery of a normal diet can effectively alleviate the symptoms. Whether the inflammation caused by other factors can be alleviated by diet requires further verification.

## Data Availability Statement

The data presented in the study are deposited in the NCBI repository, accession number is PRJNA718065.

## Ethics Statement

The animal study was reviewed and approved by Ethics Committee on laboratory animal welfare, Institute of environmental medicine and occupational medicine.

## Author Contributions 

JL and DY conceived and designed the study. RT, HD, MJ, and HW performed the experiments. ZC and YS performed the statistical analysis. JY and TC collected the samples. RT and DY wrote the paper. HL and DS reviewed and edited the manuscript. All authors contributed to the article and approved the submitted version.

## Funding

We would like to thank the key projects of the National Natural Science Foundation of China (41831287) and the Natural Science Foundation of Tianjin (19JCZDJC39900) for funding this study.

## Conflict of Interest

The authors declare that the research was conducted in the absence of any commercial or financial relationships that could be construed as a potential conflict of interest.

## References

[B1] Abbas A. K. Trotta E. D R. S. Marson A. Bluestone J. A. (2018). Revisiting IL-2: Biology and Therapeutic Prospects. Sci. Immunol. 3, 1428–1442. 10.1126/sciimmunol.aat1482 29980618

[B2] Accogli G. Crovace A. M. Mastrodonato M. Rossi G. Francioso E. G. Desantis S. (2018). Probiotic Supplementation Affects the Glycan Composition of Mucins Secreted by Brunner’s Glands of the Pig Duodenum. Ann. Anat. 218, 236–242. 10.1016/j.aanat.2018.03.008 29730471

[B3] Barcena C. Valdes-Mas R. Mayoral P. Garabaya C. Durand S. Rodriguez F.. (2019). Healthspan and Lifespan Extension by Fecal Microbiota Transplantation Into Progeroid Mice. Nat. Med. 25, 1234–1242. 10.1038/s41591-019-0504-5 31332389

[B4] Bibbo S. Ianiro G. Giorgio V. Scaldaferri F. Masucci L. Gasbarrini A.. (2016). The Role of Diet on Gut Microbiota Composition. Eur. Rev. Med. Pharmacol. Sci. 20, 4742–4749. 27906427

[B5] Bokulich N. A. Kaehler B. D. Rideout J. R. Dillon M. Bolyen E. Knight R.. (2018). Optimizing Taxonomic Classification of Marker-Gene Amplicon Sequences With QIIME 2’s q2-feature-classifier Plugin. Microbiome 6, 90. 10.1186/s40168-018-0470-z 29773078 PMC5956843

[B6] Bokulich N. A. Subramanian S. Faith J. J. Gevers D. Gordon J. I. Knight R.. (2013). Quality-filtering Vastly Improves Diversity Estimates From Illumina Amplicon Sequencing. Nat. Methods 10, 57–59. 10.1038/nmeth.2276 23202435 PMC3531572

[B7] Bolyen E. Rideout J. R. Dillon M. R. Bokulich N. A. Abnet C. C. Al-Ghalith G. A.. (2019). Author Correction: Reproducible, Interactive, Scalable and Extensible Microbiome Data Science Using QIIME 2. Nat. Biotechnol. 37, 1091. 10.1038/s41587-019-0252-6 31399723

[B8] Cao H. Liu X. An Y. Zhou G. Liu Y. Xu M.. (2017). Dysbiosis Contributes to Chronic Constipation Development *Via* Regulation of Serotonin Transporter in the Intestine. Sci. Rep. 7, 10322. 10.1038/s41598-017-10835-8 28871143 PMC5583244

[B9] Cartmell A. Munoz-Munoz J. Briggs J. A. Ndeh D. A. Lowe E. C. Basle A.. (2018). A Surface Endogalactanase in Bacteroides Thetaiotaomicron Confers Keystone Status for Arabinogalactan Degradation. Nat. Microbiol. 3, 1314–1326. 10.1038/s41564-018-0258-8 30349080 PMC6217937

[B10] Clemmensen C. Smajilovic S. Smith E. P. Woods S. C. Brauner-Osborne H. Seeley R. J.. (2013). Oral L-arginine Stimulates GLP-1 Secretion to Improve Glucose Tolerance in Male Mice. Endocrinology 154, 3978–3983. 10.1210/en.2013-1529 23959939 PMC3800753

[B11] de La Serre C. B. Ellis C. L. Lee J. Hartman A. L. Rutledge J. C. Raybould H. E. (2010). Propensity to High-Fat Diet-Induced Obesity in Rats Is Associated With Changes in the Gut Microbiota and Gut Inflammation. Am. J. Physiol. Gastrointest Liver Physiol. 299, G440–G448. 10.1152/ajpgi.00098.2010 20508158 PMC2928532

[B12] Dewar D. H. Ciclitira P. J. (2005). Clinical Features and Diagnosis of Celiac Disease. Gastroenterology 128, S19–S24. 10.1053/j.gastro.2005.02.010 15825122

[B13] Do M. H. Lee E. Oh M. J. Kim Y. Park H. Y. (2018). High-Glucose or -Fructose Diet Cause Changes of the Gut Microbiota and Metabolic Disorders in Mice Without Body Weight Change. Nutrients 10, 761–774. 10.3390/nu10060761 PMC6024874 29899272

[B14] Dorrestein P. C. Mazmanian S. K. Knight R. (2014). Finding the Missing Links Among Metabolites, Microbes, and the Host. Immunity 40, 824–832. 10.1016/j.immuni.2014.05.015 24950202 PMC4503329

[B15] Edgar R. C. (2013). UPARSE: Highly Accurate OTU Sequences From Microbial Amplicon Reads. Nat. Methods 10, 996–998. 10.1038/nmeth.2604 23955772

[B16] Erben U. Loddenkemper C. Doerfel K. Spieckermann S. Haller D. Heimesaat M. M.. (2014). A Guide to Histomorphological Evaluation of Intestinal Inflammation in Mouse Models. Int. J. Clin. Exp. Pathol. 7, 4557–4576. 25197329 PMC4152019

[B17] Furusawa Y. Obata Y. Fukuda S. Endo T. A. Nakato G. Takahashi D.. (2013). Commensal Microbe-Derived Butyrate Induces the Differentiation of Colonic Regulatory T Cells. Nature 504, 446–450. 10.1038/nature12721 24226770

[B18] Goldberg R. F. Austen W. G. Jr. Zhang X. Munene G. Mostafa G. Biswas S.. (2008). Intestinal Alkaline Phosphatase Is a Gut Mucosal Defense Factor Maintained by Enteral Nutrition. Proc. Natl. Acad. Sci. U. S. A. 105, 3551–3556. 10.1073/pnas.0712140105 18292227 PMC2265168

[B19] Gomez-Acata E. S. Centeno C. M. Falcon L. I. (2019). Methods for Extracting ‘Omes From Microbialites. J. Microbiol. Methods 160, 1–10. 10.1016/j.mimet.2019.02.014 30877015

[B20] Haas B. J. Gevers D. Earl A. M. Feldgarden M. Ward D. V. Giannoukos G.. (2011). Chimeric 16S rRNA Sequence Formation and Detection in Sanger and 454-Pyrosequenced PCR Amplicons. Genome Res. 21, 494–504. 10.1101/gr.112730.110 21212162 PMC3044863

[B21] Hamano H. Kawa S. Horiuchi A. Unno H. Furuya N. Akamatsu T.. (2001). High Serum IgG4 Concentrations in Patients With Sclerosing Pancreatitis. New Engl. J. Med. 344, 732–738. 10.1056/NEJM200103083441005 11236777

[B22] Hildebrandt M. A. Hoffmann C. Sherrill-Mix S. A. Keilbaugh S. A. Hamady M. Chen Y. Y.. (2009). High-Fat Diet Determines the Composition of the Murine Gut Microbiome Independently of Obesity. Gastroenterology 137, 1716–24 e1-2. 10.1053/j.gastro.2009.08.042 19706296 PMC2770164

[B23] Im K. Mareninov S. Diaz M. F. P. Yong W. H. (2019). An Introduction to Performing Immunofluorescence Staining. Methods Mol. Biol. 1897, 299–311. 10.1007/978-1-4939-8935-5_26 30539454 PMC6918834

[B24] Johnson A. J. Vangay P. Al-Ghalith G. A. Hillmann B. M. Ward T. L. Shields-Cutler R. R.. (2019). Daily Sampling Reveals Personalized Diet-Microbiome Associations in Humans. Cell Host Microbe 25, 789–802 e5. 10.1016/j.chom.2019.05.005 31194939

[B25] Jost T. Lacroix C. Braegger C. Chassard C. (2015). Impact of Human Milk Bacteria and Oligosaccharides on Neonatal Gut Microbiota Establishment and Gut Health. Nutr. Rev. 73, 426–437. 10.1093/nutrit/nuu016 26081453

[B26] Kanehisa M. (2019). Toward Understanding the Origin and Evolution of Cellular Organisms. Protein Sci. 28, 1947–1951. 10.1002/pro.3715 31441146 PMC6798127

[B27] Kanehisa M. Furumichi M. Sato Y. Ishiguro-Watanabe M. Tanabe M. (2020). KEGG: Integrating Viruses and Cellular Organisms. Nucleic Acids Res. 49, D545–D551. 10.1093/nar/gkaa970 PMC7779016 33125081

[B28] Kanehisa M. Goto S. (2000). KEGG: Kyoto Encyclopedia of Genes and Genomes. Nucleic Acids Res. 28, 27–30. 10.1093/nar/28.1.27 10592173 PMC102409

[B29] Kaplan G. G. (2015). The Global Burden of IBD: From 2015 to 2025. Nat. Rev. Gastroenterol. Hepatol. 12, 720–727. 10.1038/nrgastro.2015.150 26323879

[B30] Kim K. A. Gu W. Lee I. A. Joh E. H. Kim D. H. (2012). High Fat Diet-Induced Gut Microbiota Exacerbates Inflammation and Obesity in Mice Via the TLR4 Signaling Pathway. PloS One 7, e47713. 10.1371/journal.pone.0047713 23091640 PMC3473013

[B31] Kuhn K. A. Schulz H. M. Regner E. H. Severs E. L. Hendrickson J. D. Mehta G.. (2018). Bacteroidales Recruit IL-6-Producing Intraepithelial Lymphocytes in the Colon to Promote Barrier Integrity. Mucosal Immunol. 11, 357–368. 10.1038/mi.2017.55 28812548 PMC5815964

[B32] Lai N. Y. Musser M. A. Pinho-Ribeiro F. A. Baral P. Jacobson A. Ma P.. (2020). Gut-Innervating Nociceptor Neurons Regulate Peyer’s Patch Microfold Cells and SFB Levels to Mediate Salmonella Host Defense. Cell 180, 33–49.e22. 10.1016/j.cell.2019.11.014 31813624 PMC6954329

[B33] Liu R. Hong J. Xu X. Feng Q. Zhang D. Gu Y.. (2017). Gut Microbiome and Serum Metabolome Alterations in Obesity and After Weight-Loss Intervention. Nat. Med. 23, 859–868. 10.1038/nm.4358 28628112

[B34] Magoc T. Salzberg S. L. (2011). FLASH: Fast Length Adjustment of Short Reads to Improve Genome Assemblies. Bioinformatics 27, 2957–2963. 10.1093/bioinformatics/btr507 21903629 PMC3198573

[B35] Mahmud I. Sternberg S. Williams M. Garrett T. J. (2017). Comparison of Global Metabolite Extraction Strategies for Soybeans Using UHPLC-HRMS. Anal. Bioanal. Chem. 409, 6173–6180. 10.1007/s00216-017-0557-6 28844081 PMC5693640

[B36] Manichanh C. Borruel N. Casellas F. Guarner F. (2012). The Gut Microbiota in IBD. Nat. Rev. Gastroenterol. Hepatol. 9, 599–608. 10.1038/nrgastro.2012.152 22907164

[B37] Neuman M. G. (2007). Immune Dysfunction in Inflammatory Bowel Disease. Transl. Res. 149, 173–186. 10.1016/j.trsl.2006.11.009 17383591

[B38] Ngure F. M. Reid B. M. Humphrey J. H. Mbuya M. N. Pelto G. Stoltzfus R. J. (2014). Water, Sanitation, and Hygiene (WASH), Environmental Enteropathy, Nutrition, and Early Child Development: Making the Links. Ann. N. Y. Acad. Sci. 1308, 118–128. 10.1111/nyas.12330 24571214

[B39] Parada Venegas D. de la Fuente M. K. Landskron G. Gonzalez M. J. Quera R. Dijkstra G.. (2019). Short Chain Fatty Acids (Scfas)-Mediated Gut Epithelial and Immune Regulation and Its Relevance for Inflammatory Bowel Diseases. Front. Immunol. 10, 277. 10.3389/fimmu.2019.00277 30915065 PMC6421268

[B40] Pirim Gorgun E. Toker H. Korkmaz E. M. Poyraz O. (2017). IL-6 and IL-10 Gene Polymorphisms in Patients With Aggressive Periodontitis: Effects on GCF, Serum and Clinic Parameters. Braz. Oral. Res. 31, e12. 10.1590/1807-3107BOR-2017.vol31.0012 28099578

[B41] Rakoff-Nahoum S. Foster K. R. Comstock L. E. (2016). The Evolution of Cooperation Within the Gut Microbiota. Nature 533, 255–259. 10.1038/nature17626 27111508 PMC4978124

[B42] Rognes T. Flouri T. Nichols B. Quince C. Mahe F. (2016). VSEARCH: A Versatile Open Source Tool for Metagenomics. PeerJ 4, e2584. 10.7717/peerj.2584 27781170 PMC5075697

[B43] Rose F. Zeller S. A. Chakraborty T. Domann E. Machleidt T. Kronke M.. (2001). Human Endothelial Cell Activation and Mediator Release in Response to Listeria Monocytogenes Virulence Factors. Infect. Immun. 69, 897–905. 10.1128/IAI.69.2.897-905.2001 11159983 PMC97967

[B44] Sicard J. F. Le Bihan G. Vogeleer P. Jacques M. Harel J. (2017). Interactions of Intestinal Bacteria With Components of the Intestinal Mucus. Front. Cell Infect. Microbiol. 7, 387. 10.3389/fcimb.2017.00387 28929087 PMC5591952

[B45] Tain Y. L. Lee W. C. Wu K. L. H. Leu S. Chan J. Y. H. (2018). Maternal High Fructose Intake Increases the Vulnerability to Post-Weaning High-Fat Diet-Induced Programmed Hypertension in Male Offspring. Nutrients 10, 56–66. 10.3390/nu10010056 PMC5793284 29315230

[B46] Teicher I. Arlen M. Muehlbauer M. Allen A. C. (1963). The Clinical-Pathological Spectrum of Primary Ulcers of the Small Intestine. Surg. Gynecol. Obstet. 116, 196–202. 13980441

[B47] Tong M. Saito T. Zhai P. Oka S. I. Mizushima W. Nakamura M.. (2019). Mitophagy Is Essential for Maintaining Cardiac Function During High Fat Diet-Induced Diabetic Cardiomyopathy. Circ. Res. 124, 1360–1371. 10.1161/CIRCRESAHA.118.314607 30786833 PMC6483841

[B48] Wang Q. Garrity G. M. Tiedje J. M. Cole J. R. (2007). Naive Bayesian Classifier for Rapid Assignment of rRNA Sequences Into the New Bacterial Taxonomy. Appl. Environ. Microbiol. 73, 5261–5267. 10.1128/AEM.00062-07 17586664 PMC1950982

[B49] Wan Y. Wang F. Yuan J. Li J. Jiang D. Zhang J.. (2019). Effects of Dietary Fat on Gut Microbiota and Faecal Metabolites, and Their Relationship With Cardiometabolic Risk Factors: A 6-Month Randomised Controlled-Feeding Trial. Gut 68, 1417–1429. 10.1136/gutjnl-2018-317609 30782617

[B50] Weisberg S. P. McCann D. Desai M. Rosenbaum M. Leibel R. L. Ferrante A. W. Jr. (2003). Obesity is Associated With Macrophage Accumulation in Adipose Tissue. J. Clin. Invest. 112, 1796–1808. 10.1172/JCI19246 14679176 PMC296995

[B51] Wen B. Mei Z. Zeng C. Liu S. (2017). metaX: A Flexible and Comprehensive Software for Processing Metabolomics Data. BMC Bioinf. 18, 183. 10.1186/s12859-017-1579-y PMC5361702 28327092

[B52] Wilson B. C. Vatanen T. Cutfield W. S. O’Sullivan J. M. (2019). The Super-Donor Phenomenon in Fecal Microbiota Transplantation. Front. Cell Infect. Microbiol. 9, 2. 10.3389/fcimb.2019.00002 30719428 PMC6348388

[B53] Wlodarska M. Kostic A. D. Xavier R. J. (2015). An Integrative View of Microbiome-Host Interactions in Inflammatory Bowel Diseases. Cell Host Microbe 17, 577–591. 10.1016/j.chom.2015.04.008 25974300 PMC4498258

[B54] Zhang C. Zhang M. Wang S. Han R. Cao Y. Hua W.. (2010). Interactions Between Gut Microbiota, Host Genetics and Diet Relevant to Development of Metabolic Syndromes in Mice. ISME J. 4, 232–241. 10.1038/ismej.2009.112 19865183

[B55] Zhu R. Xie J. (2018). Effect of Aerobic Exercise Combined With Ginkgo Polysaccharide on Weight, Blood Glucose and Glycosylated Serum Protein in Diabetic Rats. Pak J. Pharm. Sci. 31, 1045–1050. 29731442

