# Supplementary material for: Intestinal Microbiota Mediates High-Fructose and High-Fat Diets to Induce Chronic Intestinal Inflammation
Source: Front Cell Infect Microbiol. 2021 Jun 16;11:654074. doi: 10.3389/fcimb.2021.654074 (PMC8242949; doi:10.3389/fcimb.2021.654074)
Supplement: Supplementary file 1 [file DataSheet_1.docx]

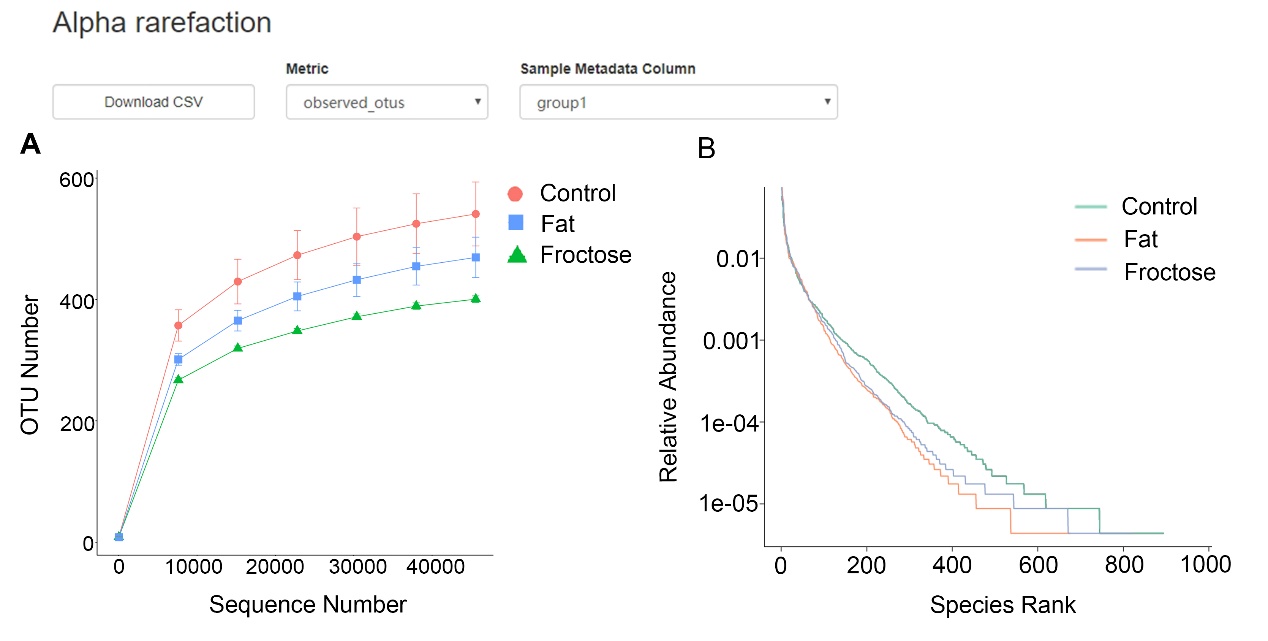


**Supplement Figure 1. Quality control of intestinal microbiota sequencing data.** Dilution curve. The abscissa shows the number of sequencing strips randomly extracted from a sample, and the ordinate is the number of operational taxonomic units (OTUs) obtained based on the number of sequencing strips (A). Rank Abundance, the horizontal axis is the ordinal number ordered by OTUs Abundance, the vertical axis is the relative Abundance of the corresponding OTUs (B).
